# Protein Substitute Absorption: A Randomised Controlled Trial Comparing CGMP vs. Amino Acids vs. Micellar Casein in Healthy Volunteers †

**DOI:** 10.3390/nu17162671

**Published:** 2025-08-19

**Authors:** Anne Daly, Alex Pinto, Sharon Evans, Tarekegn Geberhiwot, Richard Jackson, Júlio César Rocha, Jonathan C. Y. Tang, Anita MacDonald

**Affiliations:** 1 Birmingham Children’s Hospital, NHS Trust, Steelhouse Lane, Birmingham B4 6NH, UK; alex.pinto@nhs.net (A.P.); sharon.morris6@nhs.net (S.E.); anita.macdonald@nhs.net (A.M.); 2 Queen Elizabeth Hospital, NHS Trust, Birmingham B15 2GW, UK; tarekegn.geberhiwot@uhb.nhs.uk; 3 Department of Health Data Science, University of Liverpool, Liverpool L69 3GJ, UK; richj23@liverpool.ac.uk; 4 Nutrition and Metabolism, NOVA Medical School (NMS), Faculdade de Ciencias Medicas (FCM), Universidade Nova de Lisboa, 1169-056 Lisbon, Portugal; julio.rocha@porto.ac.pt; 5 CINTESIS@RISE, Nutrition and Metabolism, NOVA Medical School (NMS), Faculdade de Ciencias Medicas (FCM), Universidade Nova de Lisboa, Campo Martires da Patria, 1169-056 Lisbon, Portugal; 6 Reference Centre of Inherited Metabolic Disease, Centro Hospitalar Universitario de Lisboa Central, 1069-166 Lisbon, Portugal; 7 Comprehensive Health Research Centre (CHRC), NOVA Medical School, Faculdade de Ciencias Medicas NMS/FCM, Universidade Nova de Lisboa, 1169-056 Lisbon, Portugal; 8 Norwich Medical School, University of East Anglia, Norwich NR4 7TJ, UK; jonathan.tang@uea.ac.uk

**Keywords:** CGMP casein glycomacropeptide, amino acids, casein, phenylketonuria, absorption

## Abstract

**Background**: The rate at which amino acids (AAs) are absorbed from casein glycomacropeptide (CGMP) when given as a protein substitute in phenylketonuria (PKU) is unknown. This three-way randomised, controlled, crossover study aimed to compare the AA absorption profile of phenylalanine (Phe)-free L-amino acids (L-AAs), low-Phe CGMP (CGMP) and casein in healthy adult subjects. **Methods**: Area under the curve (AUC) was measured over 240 minutes after ingesting one dose of each protein source on three separate occasions, under the same test conditions. A total of 0.4 g/kg protein equivalent of each test product (L-AA, CGMP and casein) was given. Fasted blood samples were collected from healthy volunteers at 30, 60, 90, 120, 150, 180 and 240 minutes post-test. Insulin, blood urea nitrogen, glucose and total (TAAs), essential (EAAs), large neutral (LNAAs) and branch chain (BCAAs) amino acids were measured at each time point. **Results**: A total of 20 subjects (11 females), median age 43 y (range 23–49), with a median BMI 24.2 (20–30.5) were recruited. AUC was compared across groups. Statistically significant differences were noted for: AUC for TAAs and BCAAs between CGMP and L-AAs vs. casein [TAAs *p* = 0.008 and *p* = 0.03; BCAAs *p* = <0.001 and *p* = 0.002]. There were no AUC differences between L-AAs and CGMP. AUC was largest for L-AAs, then CGMP and finally casein. For LNAAs, EAAs, insulin, glucose and urea, there were no statistically significant differences. There was a consistent delivery of AAs for casein demonstrated by a sustained curve, but the absorption curves for L-AAs and CGMP were transient, rising rapidly and falling, with the exception of tyrosine with CGMP which showed a gradual increase over 240 minutes in contrast to L-AAs and casein. **Conclusions**: Amino acids from CGMP and L-AAs were absorbed more rapidly than casein, inferring CGMP did not mimic casein, a slow-release protein source. The tyrosine concentration curve for CGMP suggests a beneficial effect on the Phe: tyrosine ratio. Kinetic labelled studies will help bring greater understanding on the utilisation of AAs particularly important for protein synthesis.

## 1. Introduction

Phenylketonuria (PKU) is a rare, autosomal recessive, inborn error of amino acid metabolism. Deficiency of the hepatic enzyme phenylalanine hydroxylase (PAH) leads to the toxic accumulation of phenylalanine (Phe) which targets the brain and cerebrospinal fluid. Without adequate treatment, it causes severe, irreversible neurocognitive damage and behavioural and psychiatric sequalae [1,2]. The conventional treatment is a rigorous dietary Phe restriction excluding high protein foods such as meat, fish, milk, eggs, flours, breads, pasta and the sweetener aspartame (E 951), a methyl ester of the dipeptide aspartate and phenylalanine. In classical PKU, most natural protein is replaced by Phe-free L-amino acids (L-AAs) or low-Phe casein glycomacropeptide (CGMP), supplying up to 80% of nitrogen requirements. CGMP is a 64 amino acid macropeptide, naturally low in aromatic amino acids to which limiting Phe-free L-AAs (tyrosine, leucine, methionine, valine, tryptophan and histidine) are added to ensure a comprehensive intake of essential amino acids (EAAs). It contains some Phe, around 36 mg/20 g protein equivalent.

Adjunct pharmaceutical treatments include sapropterin dihydrochloride, an oral enzyme cofactor. This acts as an essential catalyst and regulator for the enzyme activity of tetrahydrobiopterin (BH_4_) [3], enabling some relaxation of natural protein restriction, but only around 20% to 40% of patients with residual enzyme activity are BH4 responsive. Pegvaliase, a pegylated bacterial-derived enzyme (phenylalanine ammonia lyase), is approved for adults aged ≥16 years and administered by injection. It leads to sustained reductions in blood Phe levels and often facilitates full liberalisation of natural protein intake [4]. Despite its efficacy, pegvaliase is associated with notable adverse effects [5] and has limited availability across Europe due to its high cost. Consequently, for individuals with classical PKU, dietary management with free/low Phe protein substitutes remains central to long-term treatment.

Free amino acids (AAs) in protein substitutes provide the essential substrates for body protein synthesis. The rate and extent of AA absorption are influenced by the type and form of dietary protein consumed, which in turn modulates postprandial protein turnover, including synthesis, breakdown and deposition. Faster absorption has been observed with whey compared to casein-based proteins [6], and notably, free AAs are absorbed significantly more rapidly than intact proteins [7]. These absorption kinetics play a key role in shaping the immediate metabolic response following consumption. Casein promotes sustained protein deposition by inhibiting catabolism without provoking excessive plasma AA concentrations, while whey accelerates protein synthesis but concurrently increases AA oxidation, reflecting its rapid bioavailability.

Understanding AA absorption kinetics is particularly important for individuals with PKU, who rely on protein substitutes as their primary source of nitrogen, essential for growth, lean body mass and broader metabolic function. In addition to supporting protein synthesis, dietary AAs play key roles in cellular signalling pathways, notably through activation of the mammalian target of rapamycin complex 1 (mTORC1), a critical regulator of protein synthesis and cell growth [8,9]. They also contribute to the biosynthesis of glutathione, creatinine and carbon skeletons needed for gluconeogenesis [10], highlighting their significance beyond structural protein requirements. Therefore, knowledge about AA absorption and retention from protein substitutes remains a critical yet under-researched area. To account for the reduced metabolic efficiency of free L-AAs, international PKU guidelines recommend a 20% higher intake of protein equivalent from protein substitutes [11]. The rate of protein digestion and AA absorption determines the postprandial rise in circulating AAs regulating postprandial muscle protein synthesis as well as AA oxidation [12], with higher AA oxidation from L-AAs compared to intact protein in patients with PKU, although this concept has been challenged. van Rijn 2007 [13] reported no differences in protein turnover in adult patients with PKU compared to healthy controls, but all subjects were given frequent meals, they had stopped growing and there was no systematic measurement of AA release over time.

The postprandial rise in plasma AA concentrations is shaped by the digestibility and absorption kinetics of the ingested protein source, as demonstrated in the foundational study by Boirie et al. [6]. Other factors that modulate postprandial muscle protein synthesis include splanchnic AA retention, the systemic availability of dietary protein-derived AAs, and the efficiency of AA delivery, uptake and intracellular signalling within muscle tissue. AA availability is primarily influenced by the rate of gastric emptying, which is affected by two key factors: the extent of milk protein coagulation in the stomach and the structural complexity of the food matrix which will change the gastric release of a protein, its digestion and AA absorption in turn changing muscle anabolic and protein turnover responses. This is illustrated with different forms of casein (sodium/calcium caseinate or micellar casein), where the metabolic properties also differ [14,15].

In non-PKU studies, several authors have studied the effect of intact protein compared to L-AAs and the effect on digestion and the speed of absorption [16]. Weijzin [17] examined protein digestion, AA absorption and the postprandial rise in AAs, demonstrating that the ingestion of a bolus of L-AAs leads to a more rapid AA absorption and greater postprandial plasma AA availability than the equivalent amount of intact milk protein, although muscle protein synthesis rates measured by Phe oxidation were similar for both protein sources. Interestingly, in this study they concluded ingestion of AAs may be preferred over ingestion of intact protein in conditions where protein digestion and AA absorption are compromised. Furthermore, oxidation rates were the same in both treatment groups which infers that L-AA sources of protein may not be inferior to whole protein.

Leucine-induced activation of mTORC1 is a central biochemical mechanism driving skeletal muscle protein synthesis following a protein-containing meal [18]. This anabolic signalling pathway is highly sensitive to postprandial leucine availability and plays a pivotal role in regulating translational initiation. In PKU, however, this meal-based anabolic response may be diminished. Natural protein sources are largely replaced by protein substitutes administered at three to four fixed intervals per day, alongside low-protein foods and a tightly controlled intake of Phe. This structured dietary pattern may alter leucine delivery kinetics and reduce mTORC1 stimulation, potentially blunting the postprandial muscle protein synthesis response.

Therefore, the magnitude and duration of post prandial dietary protein ingestion is dependent on the type of dietary protein. Identifying how to optimise the absorption and retention of AAs sourced from protein substitutes is essential, as this will influence anabolism, muscle synthesis and overall long-term growth outcomes. It is accepted that AA availability is an independent regulator of muscle protein turnover and all 20 proteinogenic AAs are required to make protein. Limitation of one or more AAs will prevent muscle protein synthesis [19]. In diets lacking mixed protein sources and relying predominantly on rapidly absorbed free AAs, both muscle and whole-body protein turnover, as well as nutrient-sensitive signalling pathways, may be adversely affected. In a previous study in children with PKU by our group, we demonstrated that CGMP (with dietary Phe modification) gave a better Phe profile compared to L-AAs. Over 24 h, the blood Phe levels were <30 μmol/L 55% of the time in the L-AAs compared to 39% in the CGMP Phe modified group. This is an important finding given all 20 AAs are essential for protein turnover and knowledge of how AAs from L-AAs and CGMP are absorbed will provide some understanding about their utilisation.

Van Calcar et al. [20] reported a significantly lower blood urea nitrogen following CGMP intake compared to L-AAs, suggesting reduced ureagenesis. However, this observation was based on a single time point at 2.5 h post-breakfast and it did not capture changes over time. The authors proposed that CGMP mimicked an intact protein due to its slower AA absorption, potentially enhancing AA retention, yet this conclusion rests on limited data. Although commercially available CGMP-based products typically contain ~70% CGMP and ~30% L-AAs [21], their absorption kinetics remain unexamined. It is also important to note that CGMP, as a small peptide, lacks the structural complexity of intact casein and would not be expected to replicate its physiological absorption profile.

To our knowledge, only one study on PKU has compared postprandial AA plasma profiles in CGMP and L-AA based protein substitutes [22]. Ahring compared the plasma AA profile of CGMP- vs. L-AAs in eight adults with PKU, evaluating the short-term effects on plasma AAs and the biomarkers insulin, blood urea nitrogen, and ghrelin but no differences were observed. No studies have compared the postprandial AA plasma profile of commercially available CGMP and L-AA protein substitutes benchmarked against a protein with a slow absorption profile such as micellar casein.

Therefore, the aims of this randomised, three-way, controlled, crossover trial was to compare the postprandial plasma AA profile of L-AAs with CGMP and micellar casein in healthy adult volunteers, systematically assessing plasma AAs, glucose, insulin and urea over 240 minutes following ingestion of each protein source. We hypothesised that plasma AA concentrations would be higher following ingestion of L-AAs compared to the two other protein sources with micellar casein showing a slow and prolonged increase of plasma AAs postprandially.

## 2. Materials and Methods

### 2.1. Subject Selection

The inclusion criteria were healthy adults aged 18–50 years, non-smokers, those not taking recreation drugs before and during the study, body mass index (BMI) between 18 and 30 kg/m^2^, willing to follow the study procedures, and able to take the study products as prescribed. Exclusion criteria included: medical conditions such as milk allergy, lactose intolerance, PKU, diabetes and renal, hepatic, cardiac or gastrointestinal disorders. Other exclusion criteria included pregnancy, lactating females, antibiotic use, donated blood or use of other medications that may affect the biomarker results within 3 months prior to study commencement.

### 2.2. Sample Size/Power Calculation

At the point of conception, there were two primary endpoints (the maximal concentration of AAs (Cmax) and area under the curve (AUC)). As the AUC was the outcome with the larger coefficient of variation, the sample size was based on this outcome. Previous data estimate the standard deviation of the AUC as approximately 50,000 umol/L, with a minimum clinically relevant difference of approximately 35,000 umol/L of total amino acids. For a Bonferroni adjusted type 1 error rate of 0.05, 20 participants were required to obtain a statistical power of 80%.

### 2.3. Study Products

Three different protein sources from commercially available products were studied: low-Phe CGMP, L-AAs and micellar casein. The CGMP (PKU Sphere, Vitaflo Ltd., Liverpool, UK) contained 78 g/100 g protein. It was a powder made into a liquid with water. CGMP was obtained via Arla with a protein content of 78–83.7% and 7–9% of sialic acid. The Phe-free L-AA supplement (PKU Cooler, Vitaflo Ltd., Liverpool, UK) was a liquid pouch containing 86 g/100 g protein; the whole protein source was micellar casein powder (Optimum Gold Standard Casein, Holland and Barrett Ltd., London, UK), containing 74 g/100 g protein. It was made into a liquid with water.

The low-Phe CGMP contained both essential and non-essential AAs. It was based on a macropeptide, supplemented with tyrosine, tryptophan, leucine, histidine and methionine, vitamins, minerals, essential fatty acids and DHA. Unlike L-AAs, it contained 36 mg/100 g of Phe. It was naturally higher in threonine and isoleucine and large neutral AAs than L-AAs (Table 1). It also contained sugar, sweetener (sucralose), soy lecithin and a thickener (E1422). E1422 is a chemically modified starch to help hold the ingredients in suspension.

The Phe-free liquid L-AAs contained essential and non-essential free AAs and was supplemented with vitamins, minerals, essential fatty acids and DHA. Ingredients included sugar, sweetener (sucralose) and an emulsifier, soy lecithin.

The casein was a flavoured powder made from 100% micellar casein (from bovine milk). It contained an emulsifier, soy lecithin, fat reduced cocoa powder flavour, salt, thickeners (cellulose, carrageenan and guar gums) and sweeteners (acesulfame potassium, sucralose). Unlike L-AAs and CGMP, it did not contain added vitamins, minerals, essential fatty acids or DHA, but did provide a source of Phe. The CGMP and L-AA sources had similar AA profiles, with some exceptions. L-AAs contained higher amounts of AAs compared to CGMP except for leucine and threonine that was higher in CGMP, and glycine and Phe was present in CGMP only (Table 1 outlines the macronutrient and amino acid composition of each product studied).

### 2.4. Study Design

**Figure 1:**
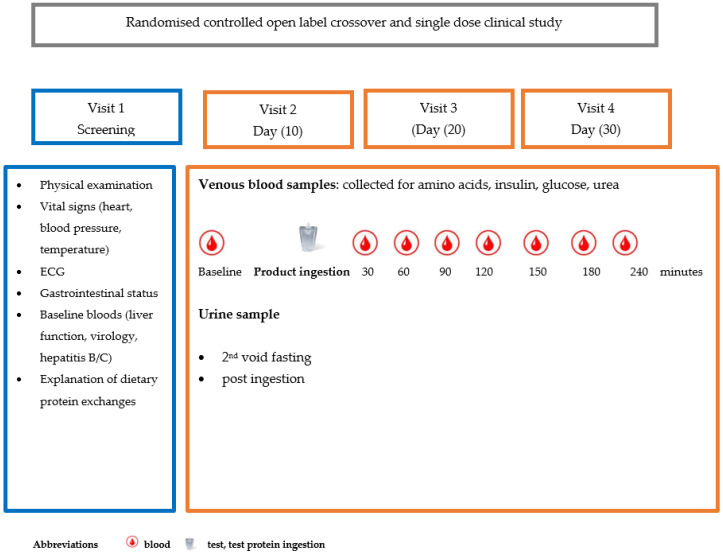
Pictorial representation of randomised controlled study design.

This was a single-centre, randomised, controlled, open-label, crossover study. It was conducted at the Clinical Research Centre at the Queen Elizabeth Hospital in Birmingham, UK. Each participant attended for 4 visits including a screening visit. The overall study time was 30 days, and each study visit had a ±7-day visit window allowing test days to be separated by a washout period of 7 to 14 days. A minimum of 1 to 4 volunteers attended at any one time, with each allocated a dedicated research nurse. Figure 1 shows the study design.

### 2.5. Screening Visit

Participant suitability was assessed by conducting a physical examination, measuring vital signs (heart rate, blood pressure, temperature) and assessment of gastrointestinal status (vomiting, constipation and diarrhoea occurrence). Anthropometric measurements (weight and height) were performed by a research nurse. Height was measured using a Seca stadiometer to the nearest 0.1 cm and weight on calibrated digital scales to the nearest 0.1 g (Seca Medical Measuring Systems and Scales, Birmingham, UK—Model 875). Medical history and medication use were recorded. A 12-lead electrocardiogram (ECG) was performed and blood tests for virology (HIV, hepatitis B and C), prothrombin time and pregnancy (in females only) were completed. Biochemical liver and kidney function were assessed. General practitioners (GPs) were informed about participant study involvement and given information about any abnormal findings.

At the screening visit, participants were given 5 g dose (as a drink) of each study product to assess their tolerability.

- Visits 2, 3 and 4

Two days prior to each study visit, participants followed a protein-controlled diet (0.83 g/kg/day) based on the WHO/FAO/UNU [23] guidelines on safe protein intake. They abstained from coffee, alcohol and strenuous exercise (e.g., running, cycling). At the screening visit, each participant was given information about estimating protein intake, including information about the protein content of common foods and interpretation of protein amounts from food labels. Each diet plan was individualised using both high and low biological protein-containing foods. Participants were provided with digital food scales and prospectively recorded all their intake in a 2-day food diary. On the evening prior to study visit 2, 3 and 4, a protein meal providing 25% of the daily safe level of protein intake for an adult (0.21 g/kg/meal) was consumed. Researchers (AP/AD) telephoned each participant the day before the study visit to confirm protein intake. This was followed by an overnight fast of 10 h. A normal unrestricted diet and exercise was maintained throughout the rest of this study.

Each participant served as their own control, and each received a standard dose of each of the study products (L-AA, CGMP and casein) based on their weight. The amount of protein equivalent given was 0.4 g/kg, providing one third of their daily protein requirement. This figure was derived from the amount suggested in the European PKU guidelines (2017) for adult patients with PKU (an increase of 40% more protein than the WHO/FAO/UNU 2007 [23] safe levels of intake). The three test products were not isocaloric, so a 50% fat emulsion (Calogen, Nutricia) was added to both the CGMP and casein sources, so all 3 study products contained the same calories for each dose administered so 28 g of protein delivered 183 Kcal. A controlled volume of water was administered with the powdered test products (CGMP and casein), with a maximum of 500 mL permitted during the first 120 minutes post-ingestion. To dissolve 5 g of powder, CGMP required 17 mL of water, whereas casein required 60 mL. No additional water was provided beyond the 120 minutes window.

Each test product gave 32.6 kcal per 5 g of protein equivalent, although their fat and carbohydrate compositions varied. All powdered products were prepared within 10 minutes in a test kitchen and consumed within a 5 minute timeframe.

### 2.6. Randomisation

Participants were randomly assigned to one of six possible sequences using a computer-generated allocation system: sequences ABC, ACB, BAC, BCA, CAB, CBA where A = CGMP; B = AAs; and C = casein. All participants sat in chairs rather than reclining on a bed as this can adversely affect gastric emptying [24].

- Intervention visits 2, 3 and 4

On participant arrival at study visits 2, 3, and 4, participants had an indwelling cannula placed in their arm by their assigned research nurse. A total of 8 blood samples were collected at each visit: baseline (pre-ingestion of the protein test product), and then at 30, 60, 90, 120,150, 180 and 240 minutes post-protein ingestion. A deviation in sample time of a maximum of ±2 minutes was permitted. Urine (for creatinine and urea) was collected twice over the study period; the first urine sample which was the second void urine of the day was collected before the protein test product ingestion and a second urine was collected during the study period from time 0 to 240 minutes. All test products had to be fully consumed within 5 minutes.

### 2.7. Measurement of Blood Biomarkers

The following plasma biomarkers were assessed at each time point: insulin, urea, blood glucose and quantitative AAs: alanine, arginine, glycine, lysine, leucine, isoleucine, Phe, methionine, threonine, serine, tryptophan, tyrosine and valine.

### 2.8. Processing of Biomarkers

Blood urea, blood glucose and urine urea were collected and analysed immediately at the research centre’s hospital laboratory. Insulin and quantitative AAs were stored until all the samples had been collected and were then analysed.

**Plasma insulin:** A minimum sample of 3.5 mL of serum was collected into a EDTA sample bottle, and allowed to clot for 30 minutes, centrifuged for 10 minutes at 3000× *g* (gravitational speed) at room temperature, and stored at −20 °C. Insulin levels were analysed at the Supra-Regional Assay Service, Peptide Hormone Laboratory, Berkshire and Surry Pathology Services, using a commercially available assay kit Iso Insulin ELISA (enzyme linked immunosorbent assay, manufactured by Mercodia (Uppsala, Sweden, GLP approved laboratory).

**Blood urea:** A minimum volume of 3 mL was collected into a lithium heparin tube and then immediately centrifuged and analysed via an Abbott Alinity c analyser. The initial rate of decrease in absorbance at 340 nm is proportional to the urea concentration in the sample.

**Blood glucose:** Samples were collected into fluoride oxalate tubes (with a minimum volume of 1 mL) and then analysed. They were centrifuged immediately and analysed by an enzymatic assay on an automated Abbott Alinity c analyser.

**Quantitative plasma amino acids:** A minimum sample of 3.5 mL of serum was collected into a EDTA sample bottle, allowed to clot for 30 minutes, centrifuged for 10 minutes at 3000× *g* (gravitational speed) at room temperature, and stored at −20 °C. They were analysed at the Bioanalytical Facility, University of East Anglia for the following AAs: alanine, arginine, glycine, lysine, leucine, isoleucine, Phe, methionine, threonine, serine, tryptophan, tyrosine and valine. They were quantified simultaneously using a Waters Xevo TQ-XS coupled with an Acquity I-class liquid chromatography tandem mass spectrometry (LC-MS/MS) system (Waters Corp., Milford, MA, USA). The LC-MS/MS system was operated in positive electrospray ionisation (ESI + ve) mode. Chromatographic separation was achieved using a Raptor Polar X column (2.1 × 100 mm, 2.7 µm) with a Raptor Polar X EXP guard column cartridge (2.7 µm, 5 mm × 2.1 mm (Restek), Bellefonte, PA, USA) maintained at a temperature of 35 °C.

Sample pretreatment was carried out by adding 50 µL of calibration standards/quality controls (Chromsystems, München, Germany) and test samples into 100 µL of 30% sulfosalicylic acid (Merck, Gillingham, UK) in water 50 µL of internal standard containing amino isotope-labelled AAs made up in 0.1 mol/L hydrochloric acid in water was added into the mixture. Each tube was vortex mixed for 30 s, then centrifuged at 10,800× *g* for 5 minutes. 10 µL of the clear supernatant was transferred into vials containing 100 µL of mobile phase B. 1 µL of the mixture was injected into the LC-MS/MS system. MassLynx version 4.2 and QuanLynx software (Waters Corp., Milford, MA, USA) were used for system control, data acquisition, baseline integration and peak quantification. The assay range was from 0.1 up to 2000 µmol/L for the AA. Inter-assay precision coefficient of variation (CV) over the 21 batches performed was between 2.1% to 7.3%.

**Urine Creatinine**: Urine creatinine samples were collected into plain universal containers and analysed on an automated Abbott Alinity c analyser. The resulting change in absorbance at 548 nm is proportional to the creatinine concentration in the sample.

### 2.9. Statistical Analysis

Continuous data were measured as median (inter-quartile range) and categorical data were presented as frequencies of counts with associated percentages. Graphical summaries using profile plots were used to visualise the data and comparisons between treatment groups were performed using Analysis of Covariance (ANCOVA). Insulin was measured on the log scale to ensure normality of model residuals. A *p*-value of 0.05 was used to determine statistical significance. All analysis was performed in R (Version 4.0).

### 2.10. Ethical Approval

This study was given favourable ethical opinion by the West Midlands Research Ethics Committee with the reference number REC reference: 20/WM/0266 and Integrated Research Application System (IRAS) number IRAS ID: 28142. It was conducted according to the principles of the ‘Declaration of Helsinki (52nd WMA General Assembly, Edinburgh, UK, 3–7 October 2000) and Good Clinical Practice Guidelines. Informed consent was obtained prior to data collection from volunteers.

## 3. Results

### 3.1. Subjects

Twenty-two participants were recruited and twenty completed this study (11 female, 9 male). One female started antibiotics and was unable to participate and one male could not be bled, despite multiple attempts at the second visit so was excluded from further participation. The median participant age was 43 years (range 23 to 49), 17 were Caucasian, 2 British Asian and 1 Afro-Caribbean. The median weight at recruitment was 69 kg (range 51.9 to 96.1), height 170 cm (range 150 to 192) and BMI 24.2 (range 20 to 30.5).

#### 3.1.1. Cmax and Tmax

The maximum concentration and time to reach maximum concentration (Cmax and Tmax, respectively) were measured for AAs, glucose and insulin. However, after analysing the data and plotting the AA values for each participant over time, it was evident that the exact Cmax and Tmax may have been undetected. This was due to the low frequency of blood sampling taken every 30 minutes whereas 15 minute sampling would have given a more comprehensive profile. Therefore, the AUC was used as a representative measure of the physiological AA pattern (Table 2).

#### 3.1.2. Area Under the Curve (Table 2)

For all the measured parameters except urea, AUC (measuring the total amount of AAs released) was largest for L-AA, then CGMP and finally casein. For urea, CGMP was highest followed by L-AAs and then casein. There were no statistically significant differences for AUC for L-AAs and CGMP. Statistically significant differences were noted for TAAs between: L-AAs and CGMP vs. casein. A significant statistical difference was also noted for BCAAs for L-AAs and CGMP vs. casein. For LNAAs, EAAs, insulin, glucose and urea there were no statistically significant differences were noted.

#### 3.1.3. Concentration of AAs at Each Time Point (TMax) (Table 3, Figure 2a–g)

CGMP and casein reached their peak concentrations for EAAs, BCAAs and LNAAs at 60 minutes except for TAAs in which casein reached the peak concentration at 30 minutes. The L-AA group reached their peak concentrations at 90 minutes, although the incremental increase of AAs between 60 and 90 minutes was only small (Table 4). Interestingly, we observed that the concentration curve for tyrosine was different between L-AA, casein and CGMP (Figure 3). The release of tyrosine showed a gradual increase over the 240 minutes in contrast to the other AAs, which showed a characteristic increase and fall. Both CGMP and L-AAs contained similar amounts of tyrosine.

**Table 3:** Mean amino acid concentrations (μmol/L) for total, branched chain, large neutral and essential amino acids from baseline to 240 minutes.

TAAs								
Time/Measure μmol/L	Baseline	30	60	90	120	150	180	240
Mean L-AA	1562	2640	3025	3027	2777	2496	2144	2108
Mean CGMP	1677	2999	3302	2988	2365	1973	1885	1695
Mean Casein	1577	2482	2475	2278	2122	2020	1871	1698
**BCAAs**								
Mean L-AA	406	966	1121	1160	1052	916	754	626
MeanCGMP	406	1117	1265	1138	838	672	593	512
Mean Casein	404	880	898	834	773	718	644	550
**LNAAs**								
Mean L-AA	748	1478	1700	1722	1564	1382	1166	1001
Mean CGMP	777	1710	1910	1725	1346	1124	1038	913
Mean Casein	761	1443	1458	1351	1264	1183	1074	923
**EAAs**								
Mean L-AA	685	1368	1555	1573	1429	1255	1047	889
Mean CGMP	710	1605	1901	1601	1216	987	901	797
Mean Casein	698	1325	1332	1230	1147	1070	970	836

**Abbreviations:** L-AA: phe free amino acids, CGMP: casein glycomacropeptide, TTAs: total amino acids, BCAAs: branched chain amino acids, LNAAs: large neutral amino acids, EAAs: essential amino acids.

**Figure 2:**
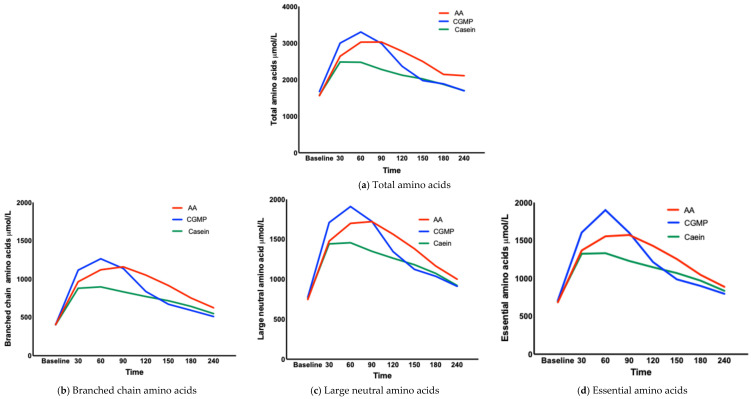

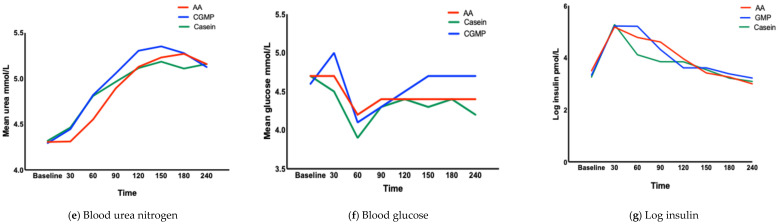
(**a**–**g**) Mean concentration curves for total, branched chain, essential and large neutral amino acids and urea, glucose and insulin.

**Table 4:** Percentage amino acid increase for TAAs, BCAAs, LNAAs and EAAs from baseline to 30 minutes compared to the total amino acids released over 240 minutes.

Amino Acid Profile	% Change from Baseline to 30 Minutes		
	L-AA	CGMP	Casein
**TAAs**	5	7	5
**BCAAs**	8	11	8
**LNAAs**	7	9	7
**EAAs**	7	9	7

**Abbreviations:** L-AA: phe free amino acids, CGMP: casein glycomacropeptide, TAA: total amino acids, BCAA: branched chain amino acids, LNAA: large neutral amino acids, EAA: essential amino acids.

**Figure 3:**
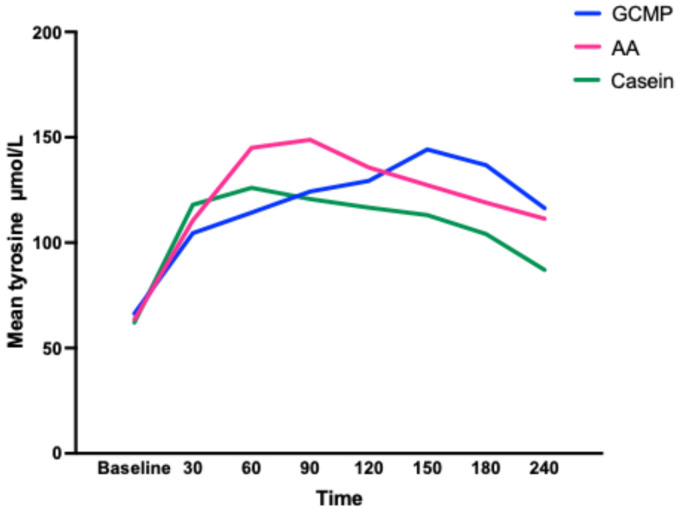
Mean concentration curves for tyrosine for L-AA, CGMP and casein.

#### 3.1.4. Change of Total, Branched Chain, Large Neutral and Essential Amino Acids Concentrations over Time (Table 4)

The magnitude of change, from each time point (e.g., baseline to 30 minutes; 30 to 60 minutes) when compared with the TAAs over 240 minutes showed a consistent pattern of increase or decrease for each test drink.

This study did not measure labelled AA release; hence, the AAs measured in the plasma may be from other endogenous sources, although a consistent pattern was noted when comparing the test drinks. The percentage of AAs measured in the plasma from baseline to 30 minutes compared to the overall release of the AAs over 240 minutes is shown in Table 4. The highest appearance of AAs for all test drinks was from baseline to 30 minutes. Both casein and L-AAs had the same release of 5% of their total AAs, while for CGMP it was higher at 7%. The AA curve for casein compared to L-AAs and CGMP from 60 to 240 minutes is also different, showing a sustained plateau of moderate hyper aminoacidemia for casein as referenced by several other researchers Ten et al. and Gorrissen et al. [25,26] but a more marked decline for CGMP and L-AAs. This slower appearance of AAs for casein compared to L-AAs and CGMP suggests a slower digestion process or alternatively greater retention by splanchnic organs leading to a smaller fall in plasma concentrations which might explain the significant differences in AUC and the differences noted when calculating time >90th centile showing a high release of AAs with CGMP.

#### 3.1.5. Time Above the 90th Centile for Total, Branched Chain, Large Neutral and Essential Amino Acids (Table 5)

The aim was to evaluate to what extent each parameter had a sustained peak (as opposed to a short-lived spike). This was assessed by examining the time period each parameter remained ≥90th centile. The time length TAAs, BCAAs, LNAAs, EAAs, insulin, urea and glucose concentrations were ≥90th centile was measured for each of the test products (Table 5). There was a statistically significant difference for the time ≥90th centile between L-AAs vs. CGMP for TAAs, LNAAs and EAAs (*p* < 0.01) and for TAAs between L-AAs vs. casein (*p* < 0.01). For TAAs, BCAAs, LNAAs and EAAs L-AAs had the greatest amount of time ≥90th centile, followed by casein and finally CGMP. Exceptions to this finding were for insulin when time ≥90th centile was greatest for L-AA, followed by CGMP and then casein; for glucose, time ≥90th centile was greatest for casein, then CGMP and finally L-AAs; and for urea, time > 90th centile was highest for CGMP, followed by L-AAs and casein.

**Table 5:** Time (range) ≥90th centile concentration for total amino acids, branched chain amino acids, large neutral amino acids, essential amino acids, insulin, glucose and urea after ingestion of test products (L-AA, CGMP and casein) over 240 minutes.

	TAA Minutes	BCAA Minutes	LNNA Minutes	EAA Minutes	Insulin Minutes	Glucose Minutes	Urea Minutes
**≥90th centile (range)**							
**L-AA**	69 (44, 69)	66 (40, 66)	72 (53, 72)	69 (45, 69)	35 (17, 35)	11 (6, 11)	36 (22, 36)
**CGMP**	25 (11, 25)	42 (31, 42)	37 (28, 37)	37 (28, 37)	19 (14, 19)	13 (9, 13)	46 (32, 46)
**Casein**	29 (12, 29)	53 (22, 53)	56 (23, 56)	57 (22, 57)	10 (8, 10)	24 (11, 24)	21 (17, 21)
**Est (95% CI); *p* value**							
**L-AA vs. CGMP**	**44**	**24**	**36**	**32**	**16**	**−2**	**−6**
	**(72, 16)**	**(53, −5)**	**(67, 5)**	**(61, 3)**	**(36, −4)**	**(−5, 1)**	**(−31, 19)**
	0.001 *	**0.050**	0.011 *	0.015 *	**0.059**	**0.079**	**0.516**
**L-AA vs.** **Casein**	**40**	**9**	**12**	**11**	**25**	**−13**	**15**
	**(65, 15)**	**(42, −24)**	**(42, −18)**	**(41, −19)**	**(38, 12)**	**(−29, 3)**	**(−13, 31)**
	0.001 *	**0.499**	**0.322**	**0.337**	<0.001 *	**0.056**	**0.964**
**CGMP vs. Casein**	**4**	**−11**	**19**	**−20**	**9**	**−2**	**8**
	**(−78, 86)**	**(−35, 13)**	**(50, −12)**	**(−55, 15)**	**(20, 1)**	**(0, −4)**	**(−16, 32)**
	**0.538**	**0.186**	**0.113**	**0.131**	0.047 *	**0.976**	**0.458**

**Abbreviations:** AUC area under the curve, TTAs: total amino acids, BCAAs: branched chain amino acids, LNAAs: large neutral amino acids, EAAs: essential amino acids. * *p* value is significant.

For insulin, the time ≥90th centile was statistically significant for L-AAs vs. casein (*p* < 0.001) and CGMP vs. casein (*p* = 0.047). This suggested the availability of more insulin for a longer period when taking L-AAs and CGMP compared to casein. For glucose the longest time ≥90th centile was for casein, then CGMP and finally L-AAs. This physiologically corresponded with insulin i.e., the longer insulin was available, the lower the glucose concentration, although there was not a statistically significant difference noted for glucose between the three test products. For urea, time above the 90th centile was longer for CGMP followed by L-AAs and casein, with no statistically significant difference noted.

The longest time ≥90th centile for TAAs, BCAAs, LNAAs, EAAs was for L-AAs, followed by casein with the shortest time for CGMP. This suggested a more rapid decline in plasma AA concentrations for CGMP compared to L-AAs and casein.

## 4. Discussion

In this three-way, randomised, crossover study, AA postprandial responses to CGMP and L-AAs were referenced against casein as a slow-release protein with a prolonged elevation of plasm AAs [27,28]. A key strength of this study was the minimisation of subject variability by employing a within-subject design, where each participant served as their own control. This approach substantially reduced inter-individual variability and enabled the evaluation of multiple interventions under controlled conditions. The rate of protein digestion and AA absorption governs the post prandial rise in circulating AAs. In this study, the pattern of hyperaminoacidemia was most pronounced following CGMP ingestion, followed by L-AAs, and was least evident with casein. The AA appearance profiles for CGMP and L-AAs differed markedly from casein, which exhibited a lower, more moderate and prolonged release pattern consistent with previous reports. These findings indicate that CGMP did not replicate the absorption kinetics of casein but instead mirrored the rapid-release profile of L-AAs, thereby refuting the initial hypothesis.

Gastric digestion plays a key role in controlling absorption kinetics impacting on nutrient bioavailability, absorption (determining the postprandial rise in circulating AAs) and physiological responses. The absorption of AAs from casein and L-AAs follow the kinetic description explained by previous researchers [17]. The AA plasma profile of CGMP has previously been described by Ahring comparing the absorption of CGMP and L-AAs in subjects with PKU, showing no significant differences for the time taken to reach peak plasma AA levels. In our study, the test drinks although having a different AA profile, were isonitrogenous. The term ‘slow’ and ‘fast’ protein describes the kinetic release of AAs from casein and whey, respectively. One is not superior to the other, both having different physiological properties. Whey protein has been shown to strongly increase muscle protein synthesis over a shorter period, while with casein, plasma AA concentrations are lower resulting in lower leucine oxidation and nonoxidative leucine disposal [6,29], with a decreased response to protein breakdown. In contrast whey, has higher nitrogen losses with reduced ability to sustained post prandial protein deposition [6].

The observed release of AAs from all three test products occurred within 30 minutes. The initial release of AAs from casein (a slow protein) was similar to L-AAs and CGMP although the concentration peak was lower and decreased over time unlike L-AAs and CMGP. Trommelen, [30], using micellar casein, observed a similar rise in plasma total AA concentrations peaking at 48 ± 38 minutes after which they declined. Boulier et al. [14] also used micellar, sodium and calcium caseinates with a similar observation. There was a significant and rapid increase in plasma EAA concentrations for all casein drinks occurring from 15 minutes. Others have reported similar findings [25,31,32], and a possible explanation is the large variability in solubility of milk protein powders (25–100%). Trommelen [30] measured the solubility of micellar casein at only 5%. This low solubility is likely to affect the rate at which the coagulum forms in the stomach leading to a more rapid protein digestion and appearance of AAs in the plasma. While solubility was not formally measured, all test products were prepared using a standardised protocol. Casein powder, however, required a greater volume of water to achieve full dispersion, indicating comparatively lower solubility under the preparation conditions applied.

In our study, with CGMP, the only individual AA that did not follow the same trend as the other AAs was tyrosine. Its curve (Figure 3) increased over the 240 minutes, reaching a maximum concentration at 150 minutes. We have no clear explanation for this observation; however, this study was conducted in healthy participants, and it remains uncertain if the same response would be observed in individuals with PKU. In practice, a sustained tyrosine level is beneficial helping to reduce the Phe:tyrosine ratio, providing greater competition at the blood–brain barrier. Ney et al. [33] made a similar observation regarding tyrosine, when measuring monoamine neurotransmitters (dopamine and serotonin) in subjects with PKU. Subjects consumed L-AAs or CGMP for 3 weeks in a randomised controlled crossover study. Intake of tyrosine and tryptophan was 50% higher when taking L-AAs, which were also consumed in larger amounts less frequently compared to CGMP. However gut plasma serotonin levels where higher in those with a PKU taking CGMP vs. L-AAs. They concluded the tyrosine from L-AAs was less bioavailable due to greater degradation by microbiota compared with the bioactive CGMP. In our own centre, when comparing CGMP with L-AAs, blood tyrosine has been consistently but not statistically significantly higher in CGMP despite similar amounts in each product, suggesting enhanced absorption [34].

Urea is the nitrogenous waste product from the catabolism of AAs, not used in biosynthetic reactions. Like Gropper et al., [16] who compared cottage cheese with L-AAs, we found no statistically significant differences in urea between the three test products. Rapid absorption of AAs is associated with greater oxidation [29] and higher blood urea nitrogen (BUN) concentrations, leading to a potential deficit in protein accretion [35]. However, there was no indication that BUN or urea were different between any of the three test products. Therefore, it was not possible to substantiate Van Calcar’s [20] research, that blood urea nitrogen was significantly lower with CGMP, suggesting reduced ureagenesis and potentially improved muscle protein synthesis compared to conventional amino acid substitutes. Verification of these effects would require dedicated kinetic analyses and direct measures of protein metabolism, such as muscle biopsy studies.

Giarratana et al. [36] used a novel physiomimic engineered protein substitute derived from Phe-free AAs, coated in sodium alginate and ethylcellulose to prolong the release of AAs in the gut. In an animal study, this was compared with conventional L-AAs, showing a prolonged release of AAs comparable to casein. In a second randomised, four-way crossover study in healthy volunteers, the engineered product was compared with a reference product, an identical product to the engineered product but uncoated, a commercial L-AA product and casein. In this study a significant difference as shown by a lower BUN, urinary urea, lower insulin peak and blood glucose was noted compared to immediate release AAs. This indicates a connection between prolonged absorption and reduced metabolic breakdown of AAs.

In our study, no significant differences between casein, L-AAs and CGMP were noted for blood glucose or insulin concentrations despite a higher carbohydrate content in L-AA and higher leucine content in L-AAs and CGMP compared with casein. Leucine may influence insulin release, glycaemic control and endocrine metabolism [37]. It has been shown that insulin secretion is positively correlated with plasma leucine, Phe and arginine concentrations [37,38]. Rasmussen et al. [39] measured the effects of leucine supplementation in elderly men and reported that plasma insulin concentration was not significantly affected due to increasing leucine intake, and although blood glucose was significantly raised, values remained within the normal parameters.

It has been suggested that peptide-based proteins may improve nitrogen retention compared to free AAs and even whole protein [40]. A systematic review by Alexander et al. [41] proposed whey-based peptide formulas facilitate optimum digestion leading to an absorptive advantage compared to free AAs and intact protein formulations. The review cited several studies suggesting that most of the nitrogen from protein is absorbed as peptides, and AAs may be absorbed more efficiently as peptides than free AAs. Peptides can enhance intestinal microcirculation improving absorption. This theory is supported by infusion studies. The transmembrane protein peptide transporter 1 (PepT1) has a well-established role as a transporter of di and tri peptides. Dietary protein and the specific AA composition of dietary protein increases the expression of Pept1 [42,43]. In previous work by our group, we demonstrated body composition and lean mass was significantly improved in children with PKU taking CGMP exclusively for 3 years compared to those on L-AA protein substitute formulations [44]. Although speculative, better absorption of peptides and improved nitrogen retention facilitated by CGMP might explain this finding. The high biological value of casein protein potentially is related to the release of peptides that have a local trophic effect on the gut and can increase mucin production having a protective effect [26].

There are several limitations to this study. We did not measure the upper anabolic threshold at which AAs were oxidised for energy or transaminated into other compounds. It is accepted protein type will influence oxidation, whey has an estimated absorption rate of 10 g/h, while cooked egg 3 g/h allowing slower absorption and greater whole-body net positive protein balance [45]. This study did not use any labelled or tracer infusion methodology hence urea was measured as a marker of amino acid waste production. CGMP is a glycomacropeptide mixed with L-AAs, it is not possible without label measurements to recognise how much of the L-AAs compared to di and tripeptides from CGMP were released altering the kinetic profile. Fat was added to CGMP and casein to ensure all products were isocaloric, and although this may have affected gastric emptying, this was unlikely given that CGMP consistently had the most rapid release of AAs. We did not measure any of the test products solubility. Therefore, it is unknown if additional vitamins and minerals or if the solubility of the casein powder affected kinetic properties. A key limitation was the 30 minutes blood sampling timeframe, which may have limited insight into the full absorption kinetics. Although prior studies, such as those by Gropper [16], have employed similar intervals, this duration may be insufficient to capture peak concentrations. More frequent blood sampling, ideally every 15 minutes would have allowed more accurate determination of Cmax and Tmax.

## 5. Conclusions

In PKU, a synthetic protein substitute, manufactured to omit Phe, is the principal form of safe protein provision to support normal growth and a wide range of nutritional and biological functions. Our study shows CGMP has the same postprandial plasma AA profile as L-AAs. Urea production was not statistically significantly different implying AAs are utilised similarly to casein. The assimilation of di and tri peptides from CGMP remains unknown and how this may be advantageously utilised needs to be answered by labelled or tracer infusion methods.

## Figures and Tables

**Table 1:** Mean nutrient and amino acid profile of L-AA, CGMP and casein, based on a mean weight of 69 kg providing 0.4 g protein equivalent per kg (28 g protein equivalent).

**Nutrient Intake per 28 g Protein Ingested**	**L-AA** **Cooler (Vitaflo Ltd.)**	**CGMP** **Sphere (Vitaflo Ltd.)**	**Casein** **(Holland and Barrett)**
Energy Kcal	183	166 + 3.8 mL calogen * 183	136 + 10.4 mL calogen * 183
Fat g	2.2	2.3 CGMP + 1.9 Calogen	0.83 Casein + 5.2 Calogen
Carbohydrate g	12.4	3.5	4.1
Protein equivalent g	28	28	28
L-phenylalanine g	-	0.052	1.35
**Amino Acid (g)**	**L-AA** **PKU Cooler (Vitaflo Ltd.)**	**CGMP** **Sphere (Vitaflo Ltd.)**	**Casein** **(Holland and Barrett)**
L-Alanine	1.29	1.18	0.78
L-Arginine	2.11	1.37	1.02
L-Aspartic acid	0.85	1.87	1.88
L-Cystine	3.29	0.35	0.10
L-Glutamine	-	3.86	5.85
Glycine	3.29	1.01	0.60
L-Histidine	1.29	1.01	0.81
L-Isoleucine	2.27	2.03	1.55
L-Leucine	3.56	4.31	2.52
L-Lysine	2.34	1.36	2.15
L-Methionine	0.63	0.40	0.76
L-Phenylalanine		0.36	
L-Proline	2.37	2.29	2.86
L-Serine	1.46	1.44	1.65
L-Threonine	2.27	3.28	1.23
L-Tryptophan	0.71	0.58	0.35
L-Tyrosine	3.34	3.22	1.00
L-Valine	2.51	1.64	1.90
L-Carnitine	0.032	-	-
Taurine	0.061	-	-
Total L-AAs excluding carnitine and taurine	33.6	31.2	27

* Calogen (Nutricia Ltd., Wiltshire, UK), a fat emulsion, was added to provide an isocaloric formula.

**Table 2:** The mean (range) for AUC for total, branched chain, large neutral and essential amino acids and glucose, insulin and urea for all three test products measured over 240 minutes.

	Test Product	AUC μmol/L minutes	Test Product	Model Est. (95% CI)	*p*-Value
TAAs	L-AA	706,116 (650,643, 706,116)	L-AA vs. CGMP	−21,401 (−104,860, 62,058)	0.617
	CGMP	659,856 (588,521, 659,856)	L-AA vs. Casein	−115,163 (−197,631, −32,695)	0.008 *
	Casein	563,634 (492,437, 563,634)	CGMP vs. Casein	21,401 (−62,058, 104,860)	0.026 *
BCAAs	L-AA	226,804 (206,987, 226,804)	L-AA vs. CGMP	−7670 (−30,666, 15,327)	0.516
	CGMP	213,680 (200,919, 213,680)	L-AA vs. Casein	−46,054 (−69,050, −23,057)	<0.001 *
	Casein	175,279 (156,044, 175,279)	CGMP vs. Casein	7670 (−15,327, 30,666)	0.002 *
LNAAs	L-AA	381,375 (351,077, 381,375)	L-AA vs. CGMP	168 (−38,958, 39,293)	0.993
	CGMP	381,062 (322,099, 381,062)	L-AA vs. Casein	−36,918 (−76,043, 2208)	0.070
	Casein	333,029 (277,647, 333,029)	CGMP vs. Casein	−168 (−39,293, 38,958)	0.068
EAAs	L-AA	355,626 (322,746, 355,626)	L-AA vs. CGMP	2054 (−36,405, 40,513)	0.917
	CGMP	353,123 (296,958, 353,123)	L-AA vs. Casein	−33,026 (−71,485, 5433)	0.098
	Casein	298,301 (255,697, 298,301)	CGMP vs. Casein	−2054 (−40,513, 36,405)	0.079
Glucose	L-AA	1034 (984, 1034)	L-AA vs. CGMP	19 (−135, 172)	0.812
	CGMP	1007 (962, 1007)	L-AA vs. Casein	−47 (−199, 105)	0.547
	Casein	1033 (948, 1033)	CGMP vs. Casein	−19 (−172, 135)	0.387
Insulin log	L-AA	1001 (880, 1001)	L-AA vs. CGMP	5824 (−9711, 21,360)	0.721
	CGMP	998 (931, 998)	L-AA vs. Casein	−2688 (−18,223, 12,847)	0.417
	Casein	937 (768, 937)	CGMP vs. Casein	−5825 (−21,360, 9711)	0.648
Urea	L-AA	1061 (983, 1061)	L-AA vs. CGMP	34 (−183, 252)	0.759
	CGMP	1113 (950, 1113)	L-AA vs. Casein	22 (−193, 237)	0.840
	Casein	1079 (891, 1079)	CGMP vs. Casein	−34 (−252, 183)	0.911

**Abbreviations:** L-AA: phe free amino acids, CGMP: casein glycomacropeptide, TAAs: total amino acids, BCAAs: branched chain amino acids, LNAAs: large neutral amino acids, EAAs: essential amino acids. * *p* = significant value.

## Data Availability

Data will be made available on request due to privacy.

## References

[1] Hillert A., Anikster Y., Belanger-Quintana A., Burlina A., Burton B.K., Carducci C., Chiesa A.E., Christodoulou J., Đorđević M., Desviat L.R. (2020). The Genetic Landscape and Epidemiology of Phenylketonuria. Am. J. Hum. Genet..

[2] van Spronsen F.J., Blau N., Harding C., Burlina A., Longo N., Bosch A.M. (2021). Phenylketonuria. Nat. Rev. Dis. Primers.

[3] Blau N. (2010). Sapropterin dihydrochloride for phenylketonuria and tetrahydrobiopterin deficiency. Expert Rev. Endocrinol. Metab..

[4] Burton B.K., Clague G.E., Harding C.O., Kucuksayrac E., Levy D.G., Lindstrom K., Longo N., Maillot F., Muntau A.C., Rutsch F. (2024). Long-term comparative effectiveness of pegvaliase versus medical nutrition therapy with and without sapropterin in adults with phenylketonuria. Mol. Genet. Metab..

[5] Hausmann O., Daha M., Longo N., Knol E., Müller I., Northrup H., Brockow K. (2019). Pegvaliase: Immunological profile and recommendations for the clinical management of hypersensitivity reactions in patients with phenylketonuria treated with this enzyme substitution therapy. Mol. Genet. Metab..

[6] Boirie Y., Dangin M., Gachon P., Vasson M.P., Maubois J.L., Beaufrere B. (1997). Slow and fast dietary proteins differently modulate postprandial protein accretion. Proc. Natl. Acad. Sci. USA.

[7] Metges C.C., El-Khoury A.E., Selvaraj A.B., Tsay R.H., Atkinson A., Regan M.M., Bequette B.J., Young V.R. (2000). Kinetics of L-[1-(13)C]leucine when ingested with free aminuteso acids, unlabeled or intrinsically labeled casein. Am. J. Physiol. Endocrinol. Metab..

[8] Kaspy M.S., Hannaian S.J., Bell Z.W., Churchward-Venne T.A. (2024). The effects of branched-chain amino acids on muscle protein synthesis, muscle protein breakdown and associated molecular signalling responses in humans: An update. Nutr. Res. Rev..

[9] Layman D.K., Anthony T.G., Rasmussen B.B., Adams S.H., Lynch C.J., Brinkworth G.D., Davis T.A. (2015). Defining meal requirements for protein to optimize metabolic roles of amino acids. Am. J. Clin. Nutr..

[10] Tome D. (2022). Efficiency of Free Amino Acids in Supporting Muscle Protein Synthesis. J. Nutr..

[11] van Wegberg A.M.J., MacDonald A., Ahring K., Belanger-Quintana A., Blau N., Bosch A.M., Burlina A., Campistol J., Feillet F., Giżewska M. (2017). The complete European guidelines on phenylketonuria: Diagnosis and treatment. Orphanet J. Rare Dis..

[12] Pennings B., Groen B., de Lange A., Gijsen A.P., Zorenc A.H., Senden J.M., Van Loon L.J.C. (2012). Amino acid absorption and subsequent muscle protein accretion following graded intakes of whey protein in elderly men. Am. J. Physiol. Endocrinol. Metab..

[13] van Rijn M., Hoeksma M., Sauer P., Szczerbak B., Gross M., Reijngoud D.J., van Spronsen F. (2007). Protein metabolism in adult patients with phenylketonuria. Nutrition.

[14] Boulier A., Denis S., Henry G., Guerin S., Alric M., Meunier N., Blot A., Pereira B., Malpuech-Brugere C., Remond D. (2023). Casein structures differently affect postprandial amino acid delivery through their intra-gastric clotting properties. Food Chem..

[15] Sakata Y., Yago T., Mori S., Seto N., Matsunaga Y., Nakamura H., Tominaga T., Miyaji K., Takeda Y. (2022). Time Courses of Gastric Volume and Content after Different Types of Casein Ingestion in Healthy Men: A Randomized Crossover Study. J. Nutr..

[16] Gropper S.S., Acosta P.B. (1991). Effect of simultaneous ingestion of L-amino acids and whole protein on plasma amino acid and urea nitrogen concentrations in humans. J. Parenter. Enter. Nutr..

[17] Weijzen M.E.G., van Gassel R.J.J., Kouw I.W.K., Trommelen J., Gorissen S.H.M., van Kranenburg J., Goessens J.P.B., van de Poll M.C.G., Verdijk L.B., van Loon L.J.C. (2022). Ingestion of Free Amino Acids Compared with an Equivalent Amount of Intact Protein Results in More Rapid Amino Acid Absorption and Greater Postprandial Plasma Amino Acid Availability Without Affecting Muscle Protein Synthesis Rates in Young Adults in a Double-Blind Randomized Trial. J. Nutr..

[18] Church D.D., Hirsch K.R., Park S., Kim I.Y., Gwin J.A., Pasiakos S.M., Wolfe R.R., Ferrando A.A. (2020). Essential Amino Acids and Protein Synthesis: Insights into Maximizing the Muscle and Whole-Body Response to Feeding. Nutrients.

[19] Bohe J., Low A., Wolfe R.R., Rennie M.J. (2003). Human muscle protein synthesis is modulated by extracellular, not intramuscular amino acid availability: A dose-response study. J. Physiol..

[20] van Calcar S.C., MacLeod E.L., Gleason S.T., Etzel M.R., Clayton M.K., Wolff J.A., Ney D.M. (2009). Improved nutritional management of phenylketonuria by using a diet containing glycomacropeptide compared with amino acids. Am. J. Clin. Nutr..

[21] Ney D.M., Stroup B.M., Clayton M.K., Murali S.G., Rice G.M., Rohr F., Levy H.L. (2016). Glycomacropeptide for nutritional management of phenylketonuria: A randomized, controlled, crossover trial. Am. J. Clin. Nutr..

[22] Ahring K.K., Lund A.M., Jensen E., Jensen T.G., Brondum-Nielsen K., Pedersen M., Bardow A., Holst J.J., Rehfeld J.F., Møller L.B. (2018). Comparison of Glycomacropeptide with Phenylalanine Free-Synthetic Amino Acids in Test Meals to PKU Patients: No Significant Differences in Biomarkers, Including Plasma Phe Levels. J. Nutr. Metab..

[23] WHO, FAO, UNU (2007). Protein and Amino Acid Requirements in Human Nutrition.

[24] Holwerda A.M., Lenaerts K., Bierau J., Wodzig W.K., van Loon L.J. (2017). Food ingestion in an upright sitting position increases postprandial amino acid availability when compared with food ingestion in a lying down position. Appl. Physiol. Nutr. Metab..

[25] Gorissen S.H.M., Trommelen J., Kouw I.W.K., Holwerda A.M., Pennings B., Verdijk L., Churchward-Venne T., Koopman R., Burd N., Fuchs C.J. (2020). Protein Type, Protein Dose, and Age Modulate Dietary Protein Digestion and Phenylalanine Absorption Kinetics and Plasma Phenylalanine Availability in Humans. J. Nutr..

[26] Ten Have G.A., Engelen M.P., Luiking Y.C., Deutz N.E. (2007). Absorption kinetics of amino acids, peptides, and intact proteins. Int. J. Sport Nutr. Exerc. Metab..

[27] Chevalier S., Gougeon R., Kreisman S.H., Cassis C., Morais J.A. (2004). The hyperinsulinemic amino acid clamp increases whole-body protein synthesis in young subjects. Metabolism.

[28] MacLeod E.L., Clayton M.K., van Calcar S.C., Ney D.M. (2010). Breakfast with glycomacropeptide compared with amino acids suppresses plasma ghrelin levels in individuals with phenylketonuria. Mol. Genet. Metab..

[29] Giordano M., Castellino P., DeFronzo R.A. (1996). Differential responsiveness of protein synthesis and degradation to amino acid availability in humans. Diabetes.

[30] Trommelen J., Weijzen M.E.G., van Kranenburg J., Ganzevles R.A., Beelen M., Verdijk L.B., van Loon L.J.C. (2020). Casein Protein Processing Strongly Modulates Post-Prandial Plasma Amino Acid Responses In Vivo in Humans. Nutrients.

[31] Pennings B., Boirie Y., Senden J.M., Gijsen A.P., Kuipers H., van Loon L.J. (2011). Whey protein stimulates postprandial muscle protein accretion more effectively than do casein and casein hydrolysate in older men. Am. J. Clin. Nutr..

[32] Traylor D.A., Gorissen S.H.M., Hopper H., Prior T., McGlory C., Phillips S.M. (2019). Aminoacidemia following ingestion of native whey protein, micellar casein, and a whey-casein blend in young men. Appl. Physiol. Nutr. Metab..

[33] Ney D.M., Murali S.G., Stroup B.M., Nair N., Sawin E.A., Rohr F., Levy H.L. (2017). Metabolomic changes demonstrate reduced bioavailability of tyrosine and altered metabolism of tryptophan via the kynurenine pathway with ingestion of medical foods in phenylketonuria. Mol. Genet. Metab..

[34] Daly A., Evans S., Chahal S., Santra S., Pinto A., Gingell C., Rocha J.C., van Spronsen F., Jackson R., MacDonald A. (2019). The Effect of Glycomacropeptide versus Amino Acids on Phenylalanine and Tyrosine Variability over 24 Hours in Children with PKU: A Randomized Controlled Trial. Nutrients.

[35] Darmaun D. (1999). Role of nutrients in the regulation of in vivo protein metabolism in humans. Acta Paediatr. Suppl..

[36] Giarratana N., Gallina G., Panzeri V., Frangi A., Canobbio A., Reiner G. (2018). A New Phe-Free Protein Substitute Engineered to Allow a Physiological Absorption of Free Amino Acids for Phenylketonuria. J. Inborn Errors Metab. Screen..

[37] van Loon L.J., Saris W.H., Verhagen H., Wagenmakers A.J. (2000). Plasma insulin responses after ingestion of different amino acid or protein mixtures with carbohydrate. Am. J. Clin. Nutr..

[38] Calbet J.A., MacLean D.A. (2002). Plasma glucagon and insulin responses depend on the rate of appearance of amino acids after ingestion of different protein solutions in humans. J. Nutr..

[39] Rasmussen B., Gilbert E., Turki A., Madden K., Elango R. (2016). Determination of the safety of leucine supplementation in healthy elderly men. Amino Acids.

[40] Nakayama K., Sanbongi C., Ikegami S. (2018). Effects of Whey Protein Hydrolysate Ingestion on Postprandial Aminoacidemia Compared with a Free Amino Acid Mixture in Young Men. Nutrients.

[41] Alexander D.D., Yan J., Bylsma L.C., Northington R.S., Grathwohl D., Steenhout P., Erdmann P., Spivey-Krobath E., Haschke F. (2016). Growth of infants consuming whey-predominant term infant formulas with a protein content of 1.8 g/100 kcal: A multicenter pooled analysis of individual participant data. Am. J. Clin. Nutr..

[42] Daniel H. (2004). Molecular and integrative physiology of intestinal peptide transport. Annu. Rev. Physiol..

[43] Freeman H.J. (2015). Clinical relevance of intestinal peptide uptake. World J. Gastrointest. Pharmacol. Ther..

[44] Daly A., Hogler W., Crabtree N., Shaw N., Evans S., Pinto A., Jackson R., Strauss B.J., Wilcox G., Rocha J.C. (2021). Growth and Body Composition in PKU Children-A Three-Year Prospective Study Comparing the Effects of L-Amino Acid to Glycomacropeptide Protein Substitutes. Nutrients.

[45] Schoenfeld B.J., Aragon A.A. (2018). How much protein can the body use in a single meal for muscle-building? Implications for daily protein distribution. J. Int. Soc. Sports Nutr..

